# Tuning the dials of Synthetic Biology

**DOI:** 10.1099/mic.0.067975-0

**Published:** 2013-07

**Authors:** James A. J. Arpino, Edward J. Hancock, James Anderson, Mauricio Barahona, Guy-Bart V. Stan, Antonis Papachristodoulou, Karen Polizzi

**Affiliations:** 1 Centre for Synthetic Biology and Innovation, Imperial College London, South Kensington Campus, London SW7 2AZ, UK; 2 Department of Life Sciences, Imperial College London, South Kensington Campus, London SW7 2AZ, UK; 3 Department of Mathematics, Imperial College London, South Kensington Campus, London SW7 2AZ, UK; 4 Department of Engineering Science, University of Oxford, Parks Road, Oxford OX1 3PJ, UK; 5 St John's College, St Giles, Oxford OX1 3JP, UK; 6 Department of Bioengineering, Imperial College London, South Kensington Campus, London SW7 2AZ, UK

## Abstract

Synthetic Biology is the ‘Engineering of Biology’ – it aims to use a forward-engineering design cycle based on specifications, modelling, analysis, experimental implementation, testing and validation to modify natural or design new, synthetic biology systems so that they behave in a predictable fashion. Motivated by the need for truly plug-and-play synthetic biological components, we present a comprehensive review of ways in which the various parts of a biological system can be modified systematically. In particular, we review the list of ‘dials’ that are available to the designer and discuss how they can be modelled, tuned and implemented. The dials are categorized according to whether they operate at the global, transcriptional, translational or post-translational level and the resolution that they operate at. We end this review with a discussion on the relative advantages and disadvantages of some dials over others.

## Introduction

The primary goal of Synthetic Biology is to create new or add additional functionality to biological systems by constructing new parts, or modifying existing biological systems (Purnick & Weiss, 2009). Central to this goal is the idea that the synthetic organism is designed following a systematic design framework with a specific objective in mind designed a priori. Ideally such design objectives can be formulated in a quantitative manner so that the performance of the designed component can be quantified and compared to the original design specification. This design framework is required both to improve reliability of individual biological components and to build functioning genetic systems with a larger number of interconnected parts (Purnick & Weiss, 2009), both considered to be current challenges of Synthetic Biology. Currently, one of the main efforts of Synthetic Biology is on building genetic systems in micro-organisms, not only because of their relative simplicity but as it is envisioned that small genetic circuits can potentially be used as a foundation for building more complex systems (Andrianantoandro *et al.*, 2006).

**Fig. 1.**
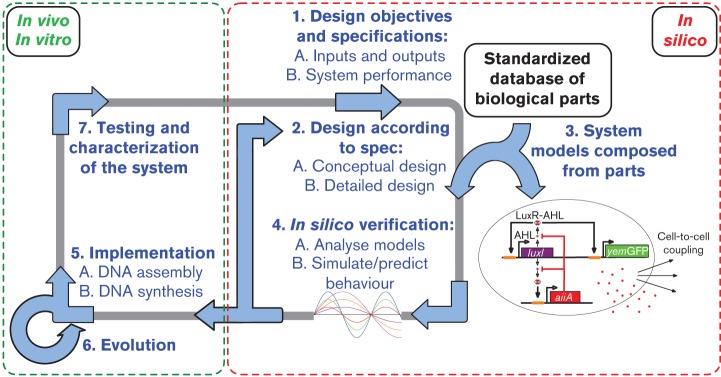
A proposed forward engineering design cycle. Steps 1–4 take place *in silico* and follow a classical engineering design approach: specification, design, modelling and analysis. Steps 5, 6 and 7 take place in the laboratory where the system is assembled, might be evolved for tuned biological function, and is characterized. The cycle can be iterated if the design does not perform to the specifications. Adapted from MacDonald *et al.* (2011).

Although Synthetic Biology has been described as the ‘Engineering of Biology’, a systematic design cycle is still not realized to its full potential, limiting the advancement of the field in terms of functionality, reliability and size of the genetic systems (Purnick & Weiss, 2009). A design framework involves design specifications, modelling, conceptual and detailed design, as well as implementation and testing (Fig. 1). In Synthetic Biology, carrying out conceptual design (e.g. choosing the basic genetic system layout) is currently relatively simple due to the limited size of present-day synthetic genetic systems, but this will become more involved as more complicated systems can be built (Purnick & Weiss, 2009; Slusarczyk *et al.*, 2012). Similarly, methods are being developed to design modules for spatial organization of the cell (Chau *et al.*, 2012; Lim *et al.*, 2012), metabolic pathways and microbial communities (Shong *et al.*, 2012). At the same time, the present design framework needs to be improved with respect to how specifications, more detailed design and robust implementation are performed. An improved forward-engineering framework would consist of a mathematical model of the system chosen in the conceptual design stage, which can provide a basis for the design, construction, characterization and testing of the developed system. The parameters in this model can then be ‘tuned’ in a systematic manner in order to ensure that the resulting model meets the design specifications. The model with the chosen parameters and predicted performance can be built and its behaviour can then guide subsequent design, implementation and testing.

However, this is easier said than done. Indeed, when ‘tuning’ the different biological dials it is important to fully understand the relationship between specifications, model parameters, biological parts and implementation in order to carry out the design process. The dials used to redesign a biological system can include tuning global parameters or transcriptional, translational and post-translational parameters in the mathematical models. Experimentally this can be achieved by using different plasmid replicons for controlling gene copy number, different promoters to control the rate of transcription initiation, different ribosome-binding sites (RBSs), or different synonymous codons for controlling translation levels or degradation rates of all the species in the systems. The models used for the basic design of genetic systems often contain parameters representing multiple biological parts and so tuning a parameter in a mathematical model can be implemented experimentally in different ways. For example, in the simplest models of gene expression, one parameter often represents many different biological characteristics, such as a ‘transcription’ parameter representing promoter strength, transcription rate and gene copy number. Each of these has different tuning ranges, uncertainties and ease of experimental modification.

In this paper, we present a comprehensive review of ways in which the various parts of a biological system can be modified systematically, focussing in particular on building genetic systems. We first discuss design and modelling of genetic systems, before reviewing in detail the typical dials that can be modified in a Synthetic Biology project. We then present various ways to tune these dials in order to achieve a desirable objective and show how tuning the parameters for each of these dials affects the output of a simple genetic system.

### System design and parameter tuning

Synthetic Biology aims to be the ‘Engineering of Biology’, where an engineering design cycle is used to systematically improve existing biological systems and create new ones (Anderson *et al.*, 2012). A traditional engineering example is the design of a chemical plant. In this case specifications may include the concentrations of the final products, a conceptual design may determine the order of processes and reactions, while a more detailed design may set variables such as concentrations and flow rates in these processes, followed by further component details based on these variables such as sizes of pipes and reaction vessels (Perry & Green, 1999). Similarly, in a biological system, the specifications may be based on protein concentrations and their response characteristics, while a conceptual design determines the layout of a genetic system needed to achieve the specifications. A more detailed design may tune some of the parameters in the mathematical model(s), such as biochemical rate constants, followed by the design of individual biological parts fulfilling these parameters such as the design of a RBS to achieve a particular translation rate. In this framework, relevant models are developed and analysed at the different design stages in order to evaluate the candidate designs and predict whether they will meet specifications. Once a detailed design is completed and verified, the system can be built and then tested to validate the design, with the design cycle repeated if at any stage the resulting functionality is not acceptable or requires improvement (RAEng, 2009).

The first step in the design of a genetic system is to specify its functionality for defined inputs and outputs. For example, the system may be required to act as a memory device or a switch (Gardner *et al.*, 2000) where the input is the concentration of an inducer and the output is the concentration of a protein. Performance specifications are required in order to determine whether the functionality is met for a particular design (Sen & Murray, 2012). These specifications can be composed of several metrics (Canton *et al.*, 2008; Del Vecchio *et al.*, 2008; Sen & Murray, 2012). For a switch, for example, there could be a requirement for the (time) mean protein concentration to be between set limits when the switch is ‘on’ or ‘off’. Retroactivity specifications, or insulation, may also be required. This ensures that the functionality of the genetic system is not negatively affected when it is connected to another system upstream or downstream (Del Vecchio *et al.*, 2008). Another metric could be the response time: the time it takes for the protein concentration to switch from low to high with a change in inducer concentration (Canton *et al.*, 2008). Also, there may be requirements on the limits of variability or noise around the mean of a protein concentration level (Lestas *et al.*, 2010). Finally, a genetic system design should meet all performance metrics despite noise and uncertainty associated with the components and chassis of the system, as well as the uncertainty in cell size due to growth.

Once specifications are set, the design of a genetic system consists of a conceptual phase (e.g. determining genetic system topology) and then using appropriate models to complete a more detailed design. The latter involves determining model parameters to meet the design specifications set. In the conceptual phase, different system topologies can be used to obtain a desired behaviour, e.g., oscillators (Purcell *et al.*, 2010; Slusarczyk *et al.*, 2012; Strelkowa & Barahona, 2010), switches (Pfeuty & Kaneko, 2009) and adaptive systems (Ma *et al.*, 2009), and more complicated systems can be built to produce more advanced behaviour (Slusarczyk *et al.*, 2012). In the detailed design phase, a mathematical model must first be built and analysed. This model will guide the design but also be used for predicting whether a proposed design meets the required specifications. The same model will also be used for steps after design such as comparison with data from the testing phase of the engineering design cycle. Such models usually take the form of differential equations based on the biochemical reactions defining the designed system (Wilkinson, 2011). These differential equations can be deterministic or stochastic. The design proceeds by using standard optimization and control engineering approaches on the deterministic models to find the best parameter choice that achieves a desired objective. A combination of both simulations (Wilkinson, 2011) and analytical methods (Murray, 2002; Tyson *et al.*, 2003) can then be used to verify the behaviour of the models. In particular, stochastic simulations are very useful in testing the variability of the system due to noise, and to ensure that stochastic effects do not substantially change the system behaviour for low biochemical species numbers (Tian & Kevin, 2006; Wilkinson, 2011). Once design parameters are selected, further models may be required for component design, such as for designing a RBS to match a tuneable parameter (Na *et al.*, 2010). The design process may need iteration, so that if no feasible choice of parameters for a particular system can meet the specifications, then a different topology can be used. Furthermore, once the system is implemented and tested, the process may need to be iterated. In particular, further detailed design or ‘tuning of the dials’ may be necessary for the circuit to function and meet specifications.

Discussion of the genetic system design leads naturally to the question of implementation, the main focus of this review. Which biological components should be modified in order to implement different genetic systems?

### A simple genetic system

**Fig. 2.**
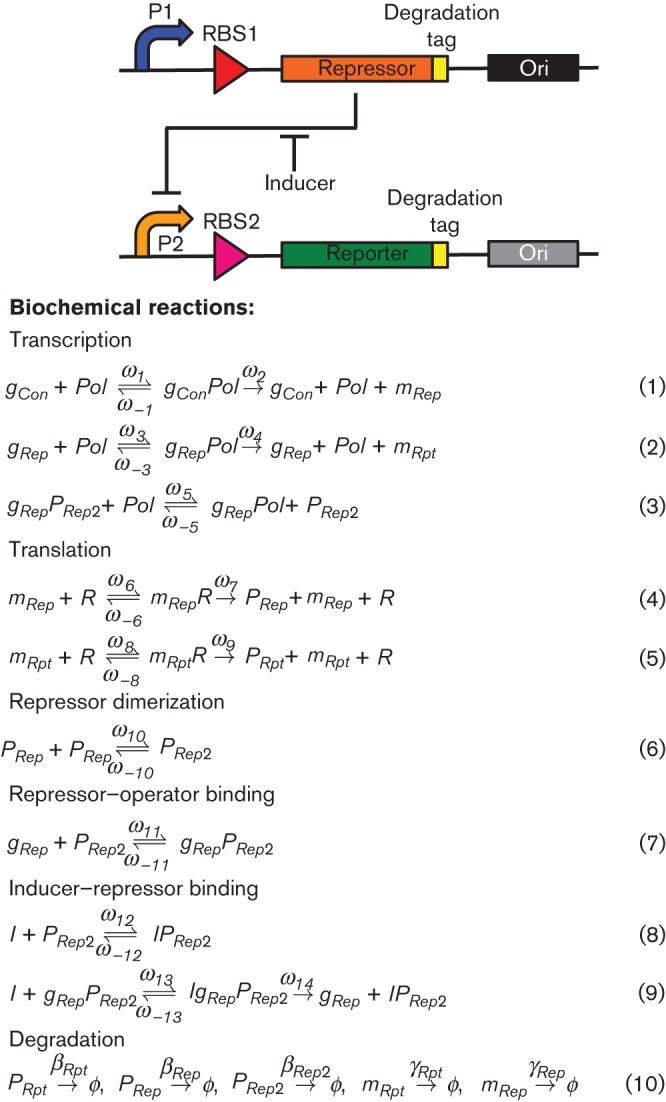
A simple genetic network with model equations. A schematic representation of a simple genetic network highlighting dials that can be tuned experimentally; promoters (P1 and P2: blue and pale orange arrows), ribosome-binding sites (RBS1 and RBS2: red and pink triangles), repressor gene (orange rectangle), reporter gene (green rectangle), protein degradation tags (yellow) and origins of replication (black and grey). Expression of the repressor results in repression of P2 whilst addition of inducer activates P2. Biochemical reactions: a set of biological reactions describing the processes that take place in the simple genetic network, where *g_Con_* is the constitutive promoter driving expression of the repressor gene, *g_Rep_* is the regulated promoter driving expression of the reporter gene, *Pol* is the RNA polymerase, *m_Rpt_*, *m_Rep_* are the reporter and repressor mRNA, *P_Rpt_*, *P_Rep_* are the reporter and repressor proteins, *I* is the inducer, *R* is the ribosome, and all other species are complexes resulting from biochemical interactions between the above listed elements. The symbol ϕ denotes degradation of a species.

Before we discuss this question, let us consider a simple, illustrative genetic system and its associated model as an example (Fig. 2). This system will be used throughout the paper to illustrate the engineering design cycle and how ‘tuning dials’ can be performed during the design and implementation stages. The example genetic system contains a constitutive promoter driving the expression of a repressor protein, which in turn represses the expression of a reporter gene from a regulated promoter. The measured output of the system is the concentration of the reporter protein while the input is the concentration of an inducer, which binds to the repressor protein thereby sequestering it away and allowing transcription initiation. The biochemical equations used to model this system are shown in Fig. 2.

The biochemical equations are the mathematical description of the underlying biochemical reactions of the system. From a biological viewpoint, the reactions that must be described are: transcription, translation, repressor–promoter and repressor–inducer interactions, and degradation of species within the system. Equations (1) and (2) describe RNA polymerase binding to a promoter followed by transcription initiation for the repressor and reporter genes, respectively. Initiation of transcription is a reversible reaction (as denoted by the double arrows and forward and reverse reaction rate constants in the equations), whereas extension is considered to be irreversible. Equation (3) is included to reflect the biological reality that most promoters have some basal level of transcription in the absence of an inducer (also called leakiness). Taken together, these equations describe the generation of mRNA species in the system.

Equations (4) and (5) describe the binding of ribosomes to a RBS on mRNA, before translation is initiated for the repressor and reporter, respectively. These steps have been described as two reactions so that the RBS strength (which is easy to modify experimentally – see below) can be accounted for separately from the translation rate, which is usually taken as a constant number of amino acids per unit time. Equations (4) and (5) together describe the rate of generation of protein species in the system.

The interactions of the repressor with the promoter and the inducer control the number of free promoters available for RNA polymerase binding. These interactions are described in equations (6)–(9). Equation (6) describes dimerization of the repressor protein, based in this example on TetR, to produce its functional form, which is capable of binding the operator region of a promoter and repressing transcription. Other repressors form different functional multimers (e.g. LacI acts as a tetramer) and would require additional equations to reflect the further multimerization steps where necessary. Equation (7) describes the binding of the functional repressor protein to the operator, while equation (8) describes inducer binding to the free repressor, which in turn prevents its binding to DNA. Equation (9) describes inducer binding to a repressor that is already bound to an operator, followed by dissociation of the inducer–repressor complex from the operator, allowing transcription to proceed.

Finally, equation (10) describes the degradation of the mRNA and protein species in the system. The degradation contributes to the steady state concentration of the species by ensuring its removal.

From this set of biochemical reactions, mass-action kinetics can be used to produce a deterministic model from the biochemical equations (Cornish-Bowden, 2004) while the chemical master equation can be used for a stochastic model (Gillespie, 1992). For the deterministic model, the mass-action kinetics can be used to describe the different reaction rates, while differential equations describe the rates of change of the concentrations due to the reactions. For the stochastic model, the equations describe the probability of a reaction occurring, e.g. an increased reaction constant makes that reaction more likely to occur at higher concentrations.

### Characterizing dials

What sets Synthetic Biology apart from traditional molecular biology is that an engineering design approach is taken to systematically design or redesign a biological system, for quantifiably improved or different functionality. This assumes that a design objective is set, against which the performance of the synthetic component will be quantified (Anderson *et al.*, 2012). To achieve the design objective, a set of biological ‘dials’ need to be tuned. In this section, we describe the set of possible dials available to the designer, and how they can be described mathematically.

### Global parameters

#### Chassis.

When designing, constructing and characterizing simple single gene expression constructs through to complex genetic networks, it is important to consider the impact of strain-to-strain variation of the behaviour of these networks. It has been shown that the abundance of RNA polymerases and ribosomes is dependent on cellular growth rates, affecting downstream processes such as transcription rate, translation rate, gene copy number, mRNA degradation, protein dilution, protein degradation and cell mass (Klumpp *et al.*, 2009). Regulated processes such as promoter repression or activation have also shown growth rate dependence, affecting genetic networks as a result (Klumpp *et al.*, 2009; Scott *et al.*, 2010; Tan *et al.*, 2009). There are a wide range of strains and organisms that can be used to harbour synthetic genetic networks, and in some cases, the networks work predictably across different strains (Prindle *et al.*, 2012) whilst in other cases the behaviour of the network can be drastically altered by changing the host cell (Egbert & Klavins, 2012). In this latter reference, two strains of *E. coli* were used that both contained a LacIΔ mutation but with slightly different genotypes, exemplifying that even similar strains can have profound effects on network function. Some tuneable elements may also be strain specific and whilst they function in one strain they may be inactive in another, e.g. the DIAL strains used to modulate plasmid copy number (Kittleson *et al.*, 2011).

#### Gene copy number.

The number of gene copies can be used to increase or decrease the amount of available protein over a partially linear range. Gene copy number can be controlled either by changing the origin of replication of a plasmid-bound gene of interest or by increasing the number of chromosomally integrated copies. Plasmid copy number has been shown to play a key role in the behaviour of genetic networks (Atkinson *et al.*, 2003; Mileyko *et al.*, 2008). The range of accessible values is limited to a maximum value that does not saturate the cellular transcription and translation machinery and does not cause undue metabolic burden on cells (‘run-away’ replication) (Nordström & Uhlin, 1992).

Gene copy number is not a continuous number. Chromosomal integration has been shown to tolerate up to five copies of the same gene (Choi *et al.*, 2006), although this could potentially be increased further, and plasmids have a few discrete values that can be accessed (Table 1). Engineering endeavours have produced plasmids with inducible copy number that can be controlled either by the binding of a ligand (Panayotatos, 1984) or through changes in temperature (Sternberg, 1990), allowing dynamic shifts in copy number to be used as a design variable. Alternatively, multiple bacterial strains have been developed (DIAL strains) that maintain the same plasmid at different steady state copy numbers (Kittleson *et al.*, 2011). These techniques give another level of control and tuneability of plasmid copy number in genetic systems.

**Table 1.** Plasmid copy number and plasmid incompatibility groups Plasmid incompatibility groups are highlighted.

Plasmid	Replicon	Copy number*	Reference
ColE1	ColE1	15–20	Schmidt & Inselburg (1982); Shizuya *et al.* (1992)
pBR322	pMB1	15–20	Balbás *et al.* (2013); Lin-Chao *et al.* (1992); Sternberg (1990)
pBR3722	pMB1	30–40†	Hakkaart *et al.* (1985); Kool & Nijkamp (1974); Lin-Chao *et al.* (1992)
pUC	pMB1	>200†	Figurski *et al.* (1979); Lin-Chao *et al.* (1992)
pACYC	p15A	18–22	Chang & Cohen (1978); Hasunuma & Sekiguchi (1979)
F1	F1	1–2	Peterson & Phillips (2008); Shizuya *et al.* (1992)
pNS358	P1 lytic *ori*	1–>25	Peterson & Phillips (2008); Sternberg (1990)
pCDF	CloDF13	10–90	Hakkaart *et al.* (1985); Kool & Nijkamp (1974); Kües & Stahl (1989)
RK2	RK2	4–7	Figurski *et al.* (1979); Kittleson *et al.* (2011)
pSC101	pSC101	~5	Hasunuma & Sekiguchi (1979); Kittleson *et al.* (2011)
pJPA12	pSC101	27	Deuschle *et al.* (1986); Gruber & Gross (2003); Peterson & Phillips (2008)
pJPA13	pSC101	~240	Becker & Hengge-Aronis (2001); Peterson & Phillips (2008)
pRSF	RSF1030	10–60	Grossman *et al.* (1987); Kües & Stahl (1989); Wang & deHaseth (2003)
pBjk2992-jtk2541	ColE2	1–60	Bernardo *et al.* (2009); Buck *et al.* (2000); Kittleson *et al.* (2011)
pBjk2993-jtk2541	R6K	5–250	Kittleson *et al.* (2011); Schleif (2000)

* Plasmid copy number is dependent on plasmid size, gene toxicity and, in some instances (indicated by †), by temperature.

The potential to maintain multiple plasmids, encoding different components from genetic networks, at different copy numbers within a cell is also possible. This is, however, dependent on the incompatibility group of the plasmid (Table 1) (Tolia & Joshua-Tor, 2006). In addition, multi-copy plasmids are maintained at a mean value in each cell, leading to population variations (Ebersbach & Gerdes, 2005) that will need to be accounted for in modelling efforts. Although plasmid copy numbers have been described here, they are dependent on the context within which they are used. The copy number of plasmids has been shown to have an inverse relationship to the plasmid size (Ebersbach & Gerdes, 2005; Zhong *et al.*, 2011) and cellular growth rate (Klumpp, 2011), and their maintenance within a cell is dependent on the toxicity of the genes encoded on the plasmid (Mileyko *et al.*, 2008).

### Transcription level design

#### Promoter type.

**Fig. 3.**
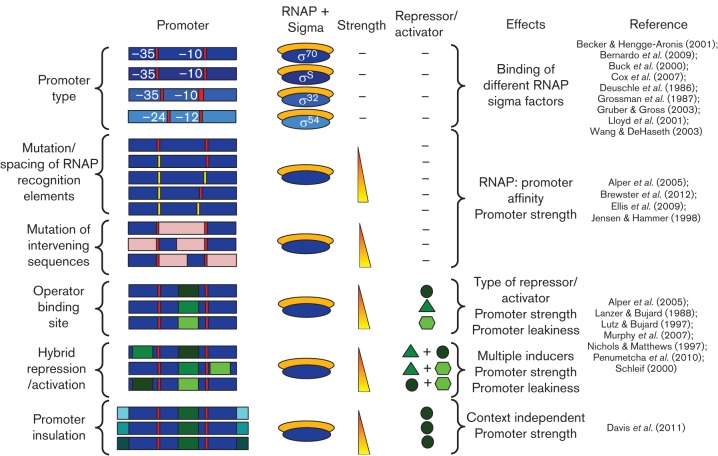
The effect of promoter architecture on promoter strength, regulation and basal transcription (leakiness). A schematic representation of different promoter architectures that can modulate the strength, regulation type and leakiness of a promoter by changing the core sigma factor (orange and blue ovals), binding sequences (blue rectangles with −35 and −10 or −24 and −12 recognition sequences in red and yellow), intervening sequences (pink), operator recognition sequences, multiplicity and location (green rectangles), repressor/activator types (green circles, triangles or hexagons) or flanking sequences (shades of turquoise).

Promoters are regions of DNA containing consensus sequences for the recruitment of the transcriptional machinery (sigma factors; RNA polymerase, RNAP) to initiate transcription (Gruber & Gross, 2003). Core promoter sequences are recognized by different sigma factors which are active under different environmental conditions, for example, *Escherichia coli* σ^70^, σ^S^, σ^32^ and σ^54^ (Table 2) (Gruber & Gross, 2003). The number of σ factors used in bacteria can vary from 1 (in *Mycoplasma genitalium*) to 63 (in *Streptomyces coelicolor*) and provides an intricate way for organisms to regulate gene expression in response to specific environmental conditions. Promoters can also be constitutively active or regulated (Fig. 3). Constitutive promoters are active without the need for any transcription factors, whilst regulated promoters have operator-binding sites for repressors or activators that block or assist RNAP binding respectively in the presence of a small molecule (inducer) or under certain environmental conditions (Lloyd *et al.*, 2001) (Table 2). Combinatorial promoter design has been implemented to generate hybrid promoters (also sometimes called logic gates) that are conditionally activated in the presence of multiple inducer signals (Fig. 3) (Cox *et al.*, 2007).

**Table 2.** Constitutive, negative, positive and hybrid regulated promoter types

Promoter	σ factor	Regulatorytype*	Regulator	Inducer	Reference
P_bla_	σ70	Con.	na	na	Deuschle *et al.* (1986); Gruber & Gross (2003)
csiD	σS	Con.	na	Starvation/hyperosmolarity	Becker & Hengge-Aronis (2001)
P_GroE_	σ32	Con.	na	Heat shock/nutrient starvation	Grossman *et al.* (1987); Wang & DeHaseth (2003)
P_o_	σ54	Con.	na	Nitrogen/nutrient starvation	Bernardo *et al.* (2009); Buck *et al.* (2000)
P_Bad_	σ70	Pos.	AraC	Arabinose	Schleif (2000)
P_LlacO-1_	σ70	Neg.	LacR	IPTG	Lutz & Bujard (1997)
P_LtetO-1_	σ70	Neg.	TetR	aTc	Lutz & Bujard (1997)
P_lac/ara-1_	σ70	Neg./Pos.	LacR/AraC	IPTG/arabinose	Lutz & Bujard (1997)

* Con., constitutive; Pos., positive regulation; Neg., negative regulation.

#### Inducer concentration.

Each transcriptional repressor/activator will respond to one or more small molecules known as inducers. There are natural inducers (e.g. allo-lactose for the Lac repressor (Lewis *et al.*, 1996) or tetracycline for the Tet repressor (Orth *et al.*, 2000)), and in some instances non-metabolizable chemical analogues that cause gratuitous induction (e.g. isopropyl-β-thiogalactoside, IPTG, for the Lac repressor (Lewis *et al.*, 1996) or anhydrotetracycline, aTc, for the Tet repressor (Lederer *et al.*, 1996)). The advantage of the chemical analogues is that their concentration level remains roughly constant. The level of transcription follows a sigmoidal response to the inducer concentration, which, over a certain range, can be approximated as linear (Table 3). Often the slope of this linear approximation is very large, which may make tuning difficult. Mutations in the small molecule binding site of the repressor could shift the range over which the response is linear (Satya Lakshmi & Rao, 2008), adding further control.

**Table 3.** Inducers and their working conditions

Promoter type	Repressor	Inducer	Working condition	Reference
Lac	LacI	IPTG	10^−4^–10^−3^ M	Lutz & Bujard (1997)
Lac	LacI*	IPTG	10^−5^–10^−4^ M	Satya Lakshmi & Rao (2008)
Lac	LacI*	Temperature	30–40 °C	Yabuta *et al.* (1995)
Tet	TetR	aTc	5–50 ng ml^−1^	Lutz & Bujard (1997)
P_BAD_	AraC	Arabinose	10^−4^–10^−1^ M	Mirasoli *et al.* (2002)
P_L_/P_R_	cI 857	Temperature	30–42 °C	Menart *et al*. (2003); Villaverde *et al.* (1993)
P_Lux_	LuxR	AHL	10^−9^–10^−6^ M	Koch (2005); Urbanowski *et al.* (2004)

* LacI mutants developed to respond to decreased IPTG concentration or alternative stimulus.

#### Promoter leakiness and basal expression.

**Fig. 4.**
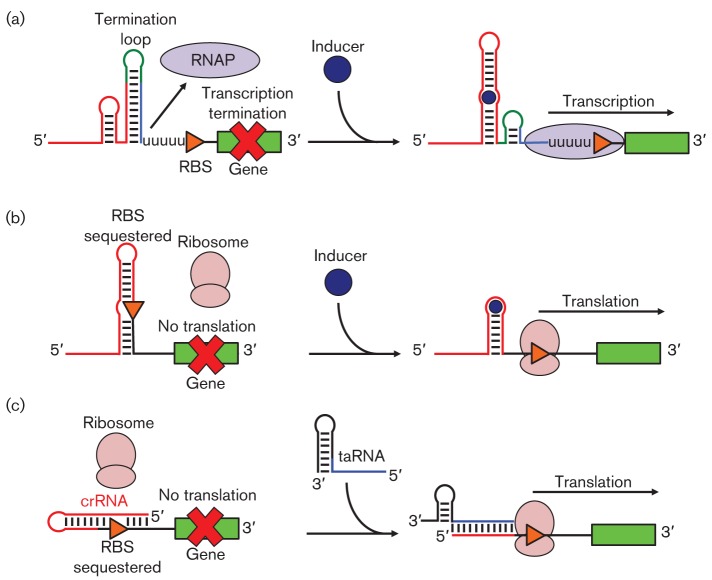
Transcriptional and translational control by riboregulators. A schematic representation of transcriptional control by a riboswitch (a), and translational control by a riboswitch (b) or a *trans*-activating RNA (taRNA) (c).

The function of promoters is often thought of encompassing two states, either on or off. However, this is very rarely the case, with transcription initiation still occurring even in the repressed state (Lanzer & Bujard, 1988). Often this behaviour is not desirable for inducible promoters. However, it can be beneficial if a particular level of basal expression is required in the repressed state but can be further increased with the addition of an inducer. The leakiness of a promoter can be tuned through the location (Lanzer & Bujard, 1988; Murphy *et al.*, 2007), number (Murphy *et al.*, 2007) and sequence (Alper *et al.*, 2005; Lanzer & Bujard, 1988) of the operator binding sites (Fig. 4) and core promoter elements. A combinatorial TetR repressed promoter library has been described previously with varying operator multiplicity and location resulting in promoters with leaky expression in the repressed state spanning more than two orders of magnitude (Murphy *et al.*, 2007). The strength of repressor binding to its operator sites can affect promoter leakiness and can be tuned through mutation of the operator DNA sequence or the DNA-binding domain of the repressor protein (Nichols & Matthews, 1997; Penumetcha *et al.*, 2010). Another way with which basal transcription can be tuned is through the co-expression of the target gene from a constitutive promoter as well as a regulated promoter, although this will also result in an increase in gene expression level in the induced state.

#### Promoter strength.

Promoter strength can be tuned by creating promoter libraries randomized in the RNAP-binding regions affecting RNAP-binding affinity (Alper *et al.*, 2005; Brewster *et al.*, 2012; Jensen & Hammer, 1998), in the operator region, which changes the strength of the interaction of the repressor/activator with the DNA (Alper *et al.*, 2005), or in the DNA sequences flanking the promoter, reducing any upstream/downstream context dependent effects on promoter strength (Davis *et al.*, 2011) (Fig. 4). Mutation of the DNA sequences between important binding motifs has also shown that a linear variation in promoter strength can be achieved (Ellis *et al.*, 2009; Jensen & Hammer, 1998). Although discrete values will be obtained through the creation of a randomized promoter library, the promoter strength can typically be treated as a continuous variable for modelling purposes. Randomized promoter libraries have elicited numerous constitutive and regulated promoters with activities spanning over two orders of magnitude (Alper *et al.*, 2005; Davis *et al.*, 2011; Ellis *et al.*, 2009; Jensen & Hammer, 1998). A limitation in the use of these promoters as predictable parts is the lack of a standardized promoter strength metric. Promoters can often perform differently from how their original characterization would suggest, due to differences in experimental conditions and measurement equipment. Therefore predicting the behaviour of a gene regulatory network component such as a promoter across different laboratories can be difficult. The need for a promoter strength metric for the accurate comparison of promoters produced from different libraries, experimental conditions and laboratories has resulted in the development of a technique to standardize a promoter strength with respect to a reference promoter, and quantifying this relative strength in terms of relative promoter units (Kelly *et al.*, 2009).

#### Placement of genes in a multi-gene construct or operon.

The length of time it takes to transcribe a gene depends upon the gene length (RNAP transcribes at a rate of ~50 bp s^−1^ (Gotta *et al.*, 1991; Vogel & Jensen, 1994)) and its distance from the transcription initiation site. Therefore, by placing one or more genes in front of the gene of interest it is possible to introduce a delay in the transcription process. It is important to note, however, that there may be unintended effects on the translation process if the secondary structure of the mRNA molecule produced causes ribosomes to disengage before translation of the gene of interest, or if secondary structure elements are introduced which alter the stability of the mRNA molecule (Carrier & Keasling, 1997a; Newbury *et al.*, 1987). In principle, this transcription delay increases linearly with the length of the superfluous genes added in front of the gene of interest and can be approximated as a continuous variable – although, strictly speaking, this is a discrete variable whose values are multiples of the time it takes to transcribe a single base (though very long mRNA constructs will tend to have larger translational effects). An increase in the length of a transcript also has a positive influence on the amount of translation from the first gene in an operon (Lim *et al.*, 2011). This is due to the fact that transcription and translation take place simultaneously in prokaryotes. Therefore, the first genes in an operon have a longer period for translation during transcription before RNAP dissociation and mRNA degradation (Lim *et al.*, 2011).

### Translation level design

#### Ribosome-binding site (RBS) strength.

The efficiency of translation initiation depends on the recruitment of ribosomes to the Shine–Dalgarno sequence, which can be stronger or weaker depending on variations in this sequence and the distance from the translation initiation codon (Chen *et al.*, 1994; Osterman *et al.*, 2013). The RBS strength is also dependent on the upstream (Komarova *et al.*, 2005) and downstream mRNA sequence (Salis *et al.*, 2009) due to the formation of local secondary structures that can influence or inhibit ribosome binding. Prediction of the strength of prokaryotic Shine–Dalgarno sequences can therefore be facilitated by the use of Chris Voigt’s simulation prediction program (RBS calculator) (Salis *et al.*, 2009) or Gyoo Yeol Jung’s UTR Designer (Seo *et al.*, 2013). Over 100 predicted RBSs have been experimentally tested showing that the translation initiation rate can be controlled over a 100 000-fold range (Salis *et al.*, 2009). The Ouyang lab used the RBS calculator to design RBSs with predicted strengths for use in a predetermined bistable toggle switch exemplifying the usefulness of this tool (Chen *et al.*, 2012). Fine-tuning of a genetic toggle switch has also been demonstrated by altering the length of the spacer between the Shine–Dalgarno sequence and the start codon (Egbert & Klavins, 2012). Comparisons of experimental data with RBS calculator predictions were in fairly good agreement dependent on the spacer sequence makeup (Egbert & Klavins, 2012).

#### Codon optimization.

Because of the degeneracy of the genetic code, it is possible to create mRNA transcripts with differing sequence that encode the same protein, eliminating rare codons and increasing translational efficiency. An altered coding sequence can also contribute to different mRNA secondary structures and, therefore, translational efficiency. Whilst standard codon optimization techniques aim to maximize protein production through using the most abundant codons observed for highly expressing native host proteins (codon adaptation index, CAI) (Angov, 2011), this technique does not take into account several factors that influence translational efficiency: translational pausing (Angov, 2011), local mRNA secondary structure (Kudla *et al.*, 2009) and tRNA abundances (Welch *et al.*, 2009). Kudla *et al.* have shown a correlation between codon optimization and the secondary structure of the mRNA at the beginning of a gene (regions −4 to +37) with the translational efficiency in *E. coli*, with a 250-fold variation in GFP expression across the constructs they tested (Kudla *et al.*, 2009). Progress has been made in predictive algorithms that take into account codon usage and tRNA abundance to optimize a gene’s coding sequence to give a desired translation efficiency (Welch *et al.*, 2009). This codon optimization algorithm could potentially be combined with RNA secondary structure prediction programs in order to facilitate a more accurate prediction in the resulting efficiency of translation.

#### mRNA decay rate.

The longevity of the mRNA transcript is controlled by its secondary structure in the un-translated regions, which protect it (Bouvet & Belasco, 1992; Carrier & Keasling, 1997b; Mackie, 2012) or make it more vulnerable (Bouvet & Belasco, 1992) to degradation by RNases, and through efficient binding and translation by ribosomes blocking RNase action (Carrier & Keasling, 1997b; Komarova *et al.*, 2005; Osterman *et al.*, 2013). The half-life for most mRNAs in *E. coli* is relatively short at ~1–2 min (Mackie, 2012). The longer-lived an mRNA molecule is, the more translation will occur from each transcript. Appending 5′ stem–loop structures of varying sizes to mRNAs has been shown to increase the mRNA half-life between 5- and 10-fold up to a half life of ~24 min (Arnold *et al.*, 1998; Hansen *et al.*, 1994). Appending 3′ REP sequences or insertion of REP sequences into intercistronic regions of polycistronic operons can also stabilize upstream mRNA transcripts by ~3-fold (Newbury *et al.*, 1987).

#### Riboregulators.

Riboswitches are RNA genetic control elements that modulate gene expression in response to an inducer molecule (Vitreschak, 2004) or transacting RNA (taRNA) (Isaacs *et al.*, 2004) without the requirement of any RNA–protein interactions. Since their discovery, a number of synthetic riboswitches have been developed that control gene expression by either premature transcriptional termination (Wachsmuth *et al.*, 2013) or by translational inhibition by sequestering RBSs (Dixon *et al.*, 2010; Lynch *et al.*, 2007; Topp *et al.*, 2010) in a dose-responsive manner to specific inducers (Fig. 4). Riboswitches that control premature transcription termination have been shown to elicit up to a 3-fold change in transcription in response to an inducer (Wachsmuth *et al.*, 2013), whilst riboswitches that modulate translation initiation have been developed that span a 2- to 150-fold range in response to an inducer. A model-directed redesign of a translational riboswitch has also been used to predictively adjust its efficiency (Beisel & Smolke, 2009). The taRNA riboregulators work by the binding of the taRNA to a *cis*-repressed mRNA (crRNA) resulting in the release of the RBS, allowing translation initiation (Isaacs *et al.*, 2004) (Fig. 4). taRNA riboregulators have been utilized in controlling a metabolic pathway and showed a ~1- to 200-fold increase in translation initiation in the presence of the trRNAs (Callura *et al.*, 2012; Isaacs *et al.*, 2004). Whilst the riboregulators described here do not require RNA–protein interactions for their function, the CRISPRi platform for transcriptional repression utilizes ribonucleoproteins (Qi *et al.*, 2013). Briefly, a small guide RNA (sgRNA) is expressed with complementary base pairing to a target DNA sequence and a secondary structural stem–loop that is recognized by a catalytically inactive RNA-binding protein, Cas9. Together the sgRNA-Cas9 ribonucleoprotein binds the target DNA sequence and inhibits initiation of transcription, elongation or transcription factor binding depending on where the sgRNA is targeted (Qi *et al.*, 2013).

### Transcriptional, translational and post-translational design

#### Inteins.

**Fig. 5.**
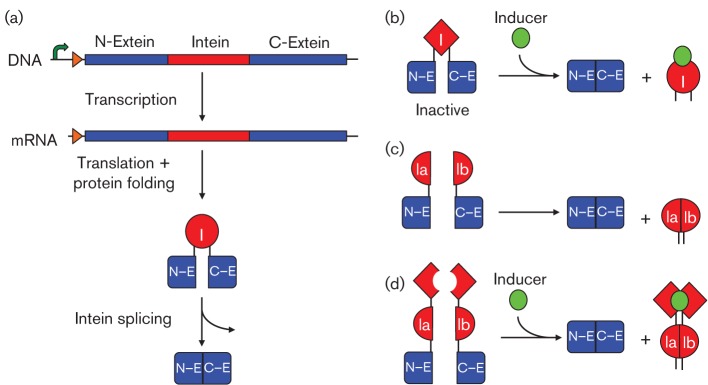
Mechanism and types of inteins. Schematic representation of the mechanism of protein splicing (a) and different intein (I) types. Small molecule (green) induced splicing (b), split intein (Ia/Ib) splicing (c) and small molecule induced split intein splicing (d).

Inteins are the protein-splicing equivalents of introns found in eukaryotic pre-mRNAs. An intein is a genetically encoded element within a target gene and is transcribed and translated together with the target protein before it undergoes autocatalytic self-excision and splicing of the target protein exteins (Gogarten *et al.*, 2002) (Fig. 5). Inteins, therefore, work at both a transcriptional and translational level by increasing the time it takes to transcribe and translate a target gene. Bacterial inteins range in size from 36 to 1986 amino acids (Perler, 2002), theoretically increasing transcription by ~2–120 s and translation by ~2–100 s. A comprehensive list of inteins and their sizes can be found at http://tools.neb.com/inbase/list_prop.php. Inteins would be beneficial for engineering delay into genetic networks, in particular tuning oscillators that depend on transcriptional and translational delay for their function (Mather *et al.*, 2009; Purcell *et al.*, 2010; Stricker *et al.*, 2008). Split inteins have also been described where the intein domain is transcribed and translated by two separate genes and the resulting proteins can undergo *trans*-splicing to produce a single functional protein (Elleuche & Pöggeler, 2010; Lockless & Muir, 2009) (Fig. 5). Post-translational control of inteins has been engineered to modulate intein splicing (Skretas, 2005), *trans*-splicing (Mootz & Muir, 2002) or a combination of both (Shi & Muir, 2005) in the presence of small molecule inducers (Fig. 5).

### Protein level design

#### Protein degradation.

The longevity of proteins within a cell can be tuned by addition of degradation tags at the N (Gur & Sauer, 2008) or the C terminus (Andersen *et al.*, 1998; Flynn *et al.*, 2001; Gur & Sauer, 2008; McGinness *et al.*, 2006) as well as internally (Gur & Sauer, 2008). Different degradation tags can be used to target proteins for degradation to one of several cellular degradation complexes (Kirstein *et al.*, 2009) (Table 4). Modifying the N-terminal residues of a protein can also target it for proteolytic degradation by the N-End rule mechanism (Erbse *et al.*, 2006; Kirstein *et al.*, 2009). Recent work has resulted in degradation tags of varying sequences that tune the rate of degradation by the ClpXP/AP degradation complexes (Andersen *et al.*, 1998; Dougan *et al.*, 2002; Flynn *et al.*, 2001; Hoskins *et al.*, 2000; McGinness *et al.*, 2006; Purcell *et al.*, 2012; Wang *et al.*, 2007). However, Cookson *et al.* have shown that an abundance of protein targeted to the ClpXP machinery can lead to a queuing effect, which, in turn, leads to a slower rate of protein degradation that is dependent on the overall concentration of tagged species in the system (Cookson *et al.*, 2011). This can be detrimental to genetic network behaviour when a fast turnaround of network elements is required but can also be beneficial in coupling separate networks through the queuing effect (Cookson *et al.*, 2011). By utilizing multiple degradation pathways, the queuing effect could potentially be suppressed.

**Table 4.** Protein degradation pathways

Protease	Cellular localization	Degradation tag/mechanism	Tag position	Approx. half-life (min)*	Reference
ClpXP	Cytoplasm	SsrA	C-terminus	10–11010–110	Andersen *et al.* (1998); Flynn *et al.* (2001); McGinness *et al.* (2006); Purcell *et al.* (2012)
ClpAP	Cytoplasm	SsrA/Rep	C-terminus	Dougan *et al* (2002); Flynn *et al.* (2001); Hoskins *et al.* (2000)
ClpAPS	Cytoplasm	N-End rule	N-terminus	2–50	Erbse *et al.* (2006); Wang *et al.* (2007)
FtsH	Inner membrane	SsrA/non-polar pentapeptide	C-terminus	2	Herman *et al.* (1998)
Tsp	Periplasm	SsrA	C-terminus	>15	Parsell *et al.* (1990); Silber *et al.* (1992)
Lon	Cytoplasm	β20	N-terminus, internal, C-terminus	>10	Gur & Sauer (2008)

* Approximate protein half-life is dependent on protein size, stability and temperature.

#### Protein activity.

Manipulation of protein activity through point mutations can be used as a means of control with a few discrete values. If the system consists entirely of genetic elements (repressors or activators), then modification of the DNA-binding affinity is best achieved by manipulating the DNA sequence, rather than attempting to mutate the protein. However, for enzymic activities, mutants with altered substrate specificity (Brannigan & Wilkinson, 2002; Wilson & Agard, 1991), kinetics (Brannigan & Wilkinson, 2002) or thermostability (Lehmann & Wyss, 2001) may already be available or can sometimes be created via protein engineering (Brannigan & Wilkinson, 2002; Lehmann & Wyss, 2001; Wilson & Agard, 1991).

### Extension to eukaryotic dials

The majority of the dials described above can also be used in eukaryotes. However, there are additional dials that can be used to tune genetic networks when working in eukaryotes, as follows.

#### Post-transcriptional modification of mRNA (splicing).

In higher eukaryotic chassis, RNA splicing can be used to introduce a delay between transcription and translation (Swinburne *et al.*, 2008). Transcripts of mRNA that have no introns will not need to be spliced, whereas those that have increasing numbers of introns will require longer transcription and processing time before translation. Splicing of mRNA can also be controlled by the addition of morpholinos (synthetic molecules that base pair with target DNA sequences) to block the pre-mRNA protein splicing machinery (Morcos, 2007). In prokaryotes, group I and II self-splicing introns have been identified, but these appear to have no known specific biological function and are thought to be remnants from an ancient RNA world (Raghavan & Minnick, 2009). Thus, with the current state of understanding, it seems they would not be very easy to target as tuneable dials at the moment.

#### Translocation.

In eukaryotes, transcription occurs in the nucleus and the resulting transcripts must then be translocated to the cytoplasm for translation (Oeffinger & Zenklusen, 2012). This will introduce a delay between transcription and translation (Grünwald & Singer, 2010). Therefore, moving a dial from a prokaryotic chassis to a eukaryotic chassis could be used as a means to introduce delay into a genetic network.

#### Protein trafficking.

In eukaryotic chassis, signal sequences are used to localize proteins to different subcellular compartments/organelles (Alberts *et al.*, 2002; Martoglio & Dobberstein, 1998). Adding a signal sequence to a protein will introduce a delay between translation and function due to the time required for transport of the protein to its intended destination (Hirschberg & Lippincott-Schwartz, 1999). This can also be used as a means to increase the effective concentration of a protein by accumulating it in a smaller volume. Protein trafficking from the cytoplasm to the inner/outer membrane and to the periplasmic space also takes place in prokaryotes, although far fewer subcellular compartments are available for sampling in prokaryotes (Driessen & Nouwen, 2008; Papanikou *et al.*, 2007).

### Discussion and perspectives

**Fig. 6.**
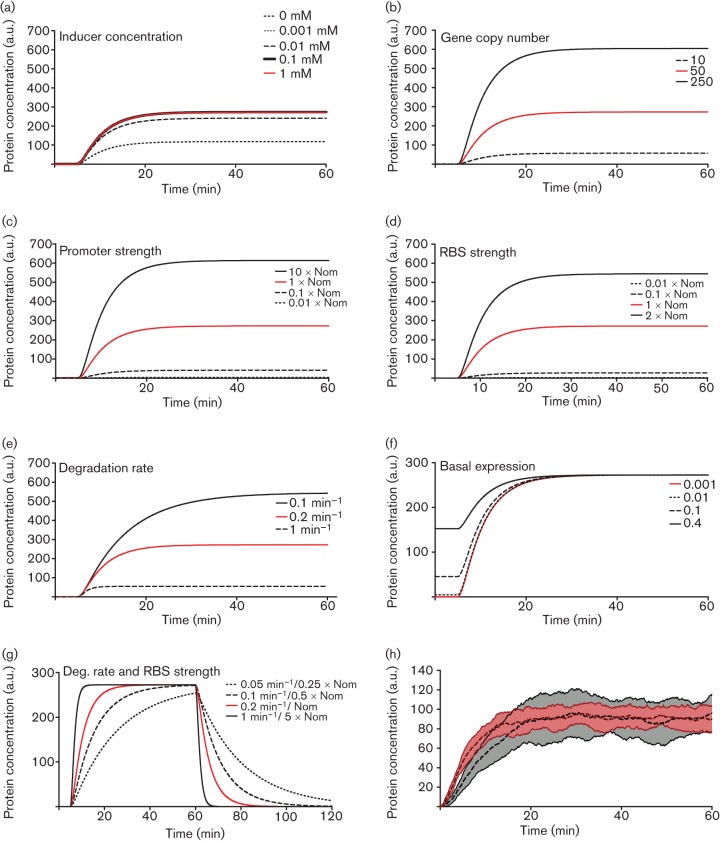
Deterministic simulations illustrating the effect of changing different dials on reporter protein concentration. The dot–dashed line in each graph gives a baseline case, which is identical across all simulations. In each panel except for (g) a single parameter has been changed whilst keeping the others constant. (a) Increasing the inducer concentration, added after 5 min, increases the steady state concentration until a saturation point is reached. (b) Increasing gene copy number increases the steady state concentration without changing the dynamics. The copy numbers used here are for real plasmid origins of replication and show that the range of values accessible using this method can be limited. Gene copy number is also a global change which affects all genes on the same plasmid equally. (c, d) Increasing or decreasing promoter or RBS strength respectively increases or decreases the steady state concentration, with respect to a nominal case (Nom), without changing dynamics. (e) Promoter leakiness increases the baseline level of protein and affects the ratio of steady state concentrations of protein in the presence or absence of inducer. Leaky promoters have a decreased dynamic range. (f) Decreasing the degradation rate increases both the steady state concentration and the time taken to reach steady state. (g) Simultaneously increasing RBS and protein degradation can change the dynamics while not modifying the steady state concentration. In this particular case the dynamics are changed, as the time taken to reach steady state is lower. Other combinations of parameters can lead to different behaviour. (h) Stochastic simulations highlighting the difference in noise between systems with high transcription and low translation (mean: dotted line, ±sd shaded light red, *n* = 25) or low transcription and high translation (mean: dashed line, ±sd shaded dark grey, *n* = 25).

Tuning parameters in mathematical models will modify the system behaviour; in this way, design specifications can be met. It is often the case, however, that a single design objective can be met via different parameter changes. For example, modifying the gene copy number, promoter strength, RBS strength, mRNA degradation rate or protein degradation rate can all change the steady state protein concentration expressed from a single gene (Fig. 6). To change the dynamics of protein accumulation, the degradation rates of the mRNA and protein can both be modified (Fig. 6) (Alon, 2007). It is, therefore, important to understand how different ‘dials’ affect the variability and noise of the system. For example, low transcription levels and high translation levels will produce a higher variability in protein concentration than high transcription and low translation levels with the same mean protein concentration at steady state (Raj & van Oudenaarden, 2008) (Fig. 6). For the case of high output variability, increasing promoter strength and decreasing RBS strength can be used to decrease variability in the output while keeping the mean output the same. Changing parameters can also modify system behaviour with regard to the output. As an example, negative feedback, either through auto-regulation or indirectly through intermediary genes in the system (Cosentino & Bates, 2012), can be used to improve robustness, reduce output variability and reduce response times of a system. Negative feedback often has trade-offs in the design and there are theoretical limits as to how much negative feedback can improve performance of genetic systems (Lestas *et al.*, 2010), which should be taken into account when tuning parameters. While simple design objectives (e.g. increasing the concentration of protein in the system) can often be designed rationally by an expert, other properties are more complex. For example, to change the system dynamics while keeping the expression level constant involves tuning multiple parameters, such as both the degradation rate and RBS strength (Fig. 6). In engineering design, this is generally achieved by setting such specifications as constraints and searching over all parameters that satisfy those constraints (Perry & Green, 1999). If more than one parameter choice meets all constraints and a benefit or cost is defined then the design can optimize the cost/benefit ratio. This is just one instance where the model can inform the choice between alternative redesigns.

The dials we have described span a range of scales and levels, and as such provide a series of options, which can be combined to achieve a design objective. Nonetheless, there are caveats: ultimately tuning any dial in a genetic network results in modulating the concentration of mRNAs and encoded proteins in the system. The dynamics of the system, however, depend on the type of control dials that are used. For example, if transcriptional level control dials (regulated promoters, transcriptional riboswitches) are used there will be a longer delay before a functional protein is produced since transcription, translation and protein folding must take place sequentially. On the other hand, if translational or post-translational control dials are used, the functional protein will be produced in a shorter period of time. Therefore if a genetic network with fast dynamics is required, it may be beneficial to factor post-translational control into the design process rather than transcriptional control. There will typically be a trade-off between the cell producing high levels of protein, poised to carry out its function, and gene expression inducing metabolic burden on the cell. The use of degradation tags on proteins also incurs a high metabolic burden since it decreases the steady state concentration by increasing the protein turnover rate. This results in resources being used to make a protein that is then targeted for fast degradation and is thus short-lived.

Linking multiple dials together can provide a genetic network with several avenues for tuning, providing a high level of control over network behaviour, e.g. coarse tuning through different origins of replication (modulating gene copy number), medium-level tuning through different promoters, and fine-tuning with different RBSs. However, linking different dials together often takes them out of the context under which they were initially characterized, thereby reducing the predictability of their individual behaviour and of their impact on the designed systems. For instance, it has been shown that increasing gene copy number can reduce the dynamic range and increase the leakiness of a promoter (Lutz & Bujard, 1997). Leakiness could potentially be reduced by adding a second layer of transcriptional or translational control by the addition of a third tuneable element, a riboregulator, thus combating the unwanted effects of copy number on promoter behaviour. Addition and removal of control dials from a genetic network can be experimentally facilitated by the use of modular plasmid designs with large multiple cloning sites, allowing for the sequential addition of network components. Litcofsky *et al.* demonstrated this by constructing a simple toggle switch and a three-node or four-node feed-forward loop (Litcofsky *et al.*, 2012). Progress has also been made in the use of bio-parts in a plug-and-play methodology through the standardization of plasmid design (Silva-Rocha *et al.*, 2013).

Another factor to keep in mind is that, experimentally, some dials are easier to predictably tune than others. Altering gene copy number can be easy to achieve by replacing the origin of replication on plasmid-borne genetic networks or through single or multiple genomic integrations. Whilst the gene copy number can be controlled exactly through genomic integration, plasmid copy numbers can be harder to tune to exact levels given that many factors, described above, can affect plasmid copy numbers. Cell chassis tuning is less simple, potentially requiring genome engineering to achieve particular cell traits that impact on genetic network behaviour. As the effects of different cell chassis on network behaviour are currently not predictable, two approaches are available to aid in network redesign: (1) a genetic network can be characterized in several cell chassis to envisage the differential effects on the network with alternate chassis environments or (2) by using software such as Intermine (Smith *et al.*, 2012) or Ondex (Köhler *et al.*, 2006), developed for searching, data mining and integration of biological databases, which could help in identifying particular characteristics of different cell chassis to help direct and inform the design process. While the use of *in silico* approaches to design RBSs with predicted strengths can speed up the design and tuning process (Salis *et al.*, 2009), tuning most other dials can be time intensive due to the lack of software to help predict the effect changes on these dials may have. For example, whilst new promoters can be engineered, as described previously, there is often a trade-off between promoter strength, repressor strength, dynamic range and leakiness (Lanzer & Bujard, 1988). Trying to tune one of these parameters can often alter the others. Therefore, predictively designing a promoter with specific attributes is not straightforward. However, these trade-offs are common in engineering design for other fields, where they are typically handled using an optimization framework which considers various constraints and objective functions in the design (Boyd & Vandenberghe, 2004; Perry & Green, 1999; Dolan *et al.*, 2012). Directed evolution approaches (Lutz & Patrick, 2004; Neylon, 2004) are available to produce libraries of promoters but they often require extensive screening for desired characteristics and are thus often experimentally time consuming. Likewise, adding transcriptional level control with riboswitches can be relatively easy, whilst using a riboswitch for translational level control is more difficult as its function is often dependent on the RBS sequence, which cannot be easily tuned without affecting the riboswitch integrity.

Two of the pioneering hallmarks for Synthetic Biology were the realization of simple designs inspired by existing electronic counterparts, i.e. a genetic toggle switch (Gardner *et al.*, 2000) and an oscillator (Stricker *et al.*, 2008). Their designs were inspired by a model-guided approach that provided an *in silico* assessment of the qualitative behaviour of these simple genetic networks. Further advancements in the field led to the use of a model-guided design (Ellis *et al.*, 2009), which allowed for the tuning of transcriptional layer dials (promoter characteristics) in a reliable and relatively straightforward manner, to achieve a predictable genetic timer that controls yeast sedimentation (Ellis *et al.*, 2009). Within the scope of cell-based biosensing, model-guided design approaches have been used to inform the development of layered AND gates, housed in separate cell populations, which communicate through quorum sensing to detect specific combinations of metals (Beguerisse-Díaz *et al.*, 2012; Wang *et al.*, 2013). One of the most complex genetic designs achieved to date is exemplified by Moon *et al.* (2012), who used a combination of computational tools, model-guided design and directed evolution to construct a four-input AND gate that consists of three circuits integrating four inducible systems within a single *E. coli* cell.

In order to realize the need for truly plug-and-play Synthetic Biology, the designer has to appreciate the types of dials they can use to achieve their design objectives: some are ‘difficult to tune’, some are ‘sensitive’ and some others are ‘uncertain’. In this review, we have described some of the possible dials that are available to the Synthetic Biologist at various organizational layers, thus opening the possibility for a design cycle that will involve mathematical modelling and optimization to produce systems with predictable, robust behaviour.
